# Opinion: regulatory genotoxicity: past, present and future

**DOI:** 10.1186/s41021-022-00242-5

**Published:** 2022-04-22

**Authors:** Makoto Hayashi

**Affiliations:** makoto international consulting, 4-23-3-1, Ebina, Kanagawa 243-0431 Japan

**Keywords:** Genotoxicity, Regulatory science, Evaluation and interpretation, Statistical evaluation

## Abstract

I will reflect on the role of genotoxicity in the regulation of chemical safety, summarizing the past and current situation, and giving personal views for the future. This includes how genotoxicity information has been, and is being, used in the evaluation of the safety of chemical substances including pharmaceuticals, pesticides, food additives and industrial chemicals before they are introduced into the market for sale.

In Japan, the Industrial Safety and Health Act, enacted in 1972, assures workers’ safety by including safety assessment of chemicals to which workers may be exposed in the workplace. The law firstly included the bacterial gene mutation assay with rat liver microsome fraction (Ames test) for the evaluation of chemical mutagenicity to predict carcinogenic potential, which was the forerunner of requiring a genotoxicity test by law. Since then, genotoxicity, especially the Ames test and the in vitro chromosomal aberration test using cultured mammalian cells (especially Chinese hamster cells) have been incorporated into several laws to assess the safety of various chemicals. Many test systems for different endpoints have been developed, improved, and used in practice. The battery strategy, combining several test systems to detect as many genotoxic chemicals as possible, was implemented because no one test system can detect all genotoxic agents with different mechanisms of genetic damage. In general, the standard battery consists of the Ames test, in vitro chromosomal aberration test and the in vivo rodent erythrocyte micronucleus test as a representative in vivo assay. Many other test systems have been used for supplementary testing as well as for research studies. Important keywords for regulatory science include 1) guidelines, 2) Good Laboratory Practice, 3) evaluation and interpretation of test results. Here, I discuss on these key points, and give personal opinions for the future.

## Introduction

Regulatory genotoxicology is defined as genotoxic studies to support regulatory decision-making related to chemical safety. I discuss briefly the necessity of guidelines, Good Laboratory Practice (GLP), and more deeply the evaluation and interpretation of genotoxic assay results for supporting rational judgement. In Japan, mutagenicity testing started when the bacterial gene mutation assay with the rat liver microsome fraction (Ames test) [1], was introduced into law: The Industrial Safety and Health Act, required mandatory testing when a new chemical was proposed to be manufactured as part of safety assessment especially for prediction of the carcinogenic potential of the chemical. Based on the good correlation between chemical carcinogenicity and mutagenicity, the mutation assay had been expected to be a good biomarker of potential carcinogenicity. Subsequently, chromosomal aberration and DNA damage were added as additional endpoints of genotoxicity and became widely used for genotoxic evaluation [2, 3].

Genotoxicity test systems started mainly with in vitro microorganisms ( bacteria and yeast in/on agar in plates) and cultured mammalian cells, and in vivo test systems such as the rodent micronucleus test using bone marrow or blood cells were subsequently introduced [4, 5]. Several test systems were included the OECD test guidelines, of which many have been revised and some were deleted and archived separately.

## Past and present

In the first era, as I mentioned above, the Ames test was thought to be a good tool for prediction of chemical carcinogenicity. This assay was followed by the in vitro chromosomal aberration assay, using mainly Chinese hamster cultured cells to detect chromosomal aberrations as another major endpoint of mutagenicity.

The terms “genotoxicity” and “mutagenicity” have been not well defined and have been used interchangeably sometimes and defined differently at other times. Most frequently, “mutagenicity” is meant to apply only to gene mutation and chromosomal aberration while “genotoxicity” has a wider coverage including DNA damage, sister-chromatid exchange, and other genetic events in addition to mutagenicity [6]. Here I use mainly “genotoxicity” to refer to a wider range of meaning, while I would like to use “mutagenicity” to restrict the meanings of gene mutation and chromosomal structural aberration.

Many studies have been conducted to detect genotoxic chemicals in order to predict carcinogenicity. Therefore, the sensitivity of the test system was considered important in order to avoid false-negative results while false-positive results often received too little attention. The fair balancing of these two “errors” made difficult to construct a rational battery system for the detection of genotoxicity.

In the second era, attempts were made to standardize the methods and established international and domestic guidelines for assessing genotoxicity. This included establishing OECD test guidelines as the international standard for test protocols. Most of the voices demanded transparency and fairness when evaluating the test results, and we believed that the guidelines were authoritative and well-constructed. Nevertheless, it is equally important to conduct the tests according to the guidelines. The same discussion was held regarding GLP, which was triggered by the identification of regulatory studies at contract laboratories that were faked or not clearly documented, and many countries, including Japan, decided to introduce GLP’s for international harmonization (for example; OECD GLP [7]). This resulted in the Safety Guidelines established through the International Conferences on Harmonization (ICH) for human use of pharmaceutical drugs [8–10]. These guidelines and GLP have played important roles producing test data of high quality for regulatory use to judge the safety of chemical substances. The above body of testing guidelines has become an indispensable part of the implementation of safety tests.

## Evaluation and interpretation of genotoxicity data

Genotoxicity information has been widely used for the safety risk assessment of chemicals together with general toxicity, carcinogenicity, reproductive toxicity, and other endpoints. The different feature of genotoxicity from many other toxicities is visibility of toxicological phenomena. Although many genetic diseases and chromosomal anomalies are well known, most of them are caused by heredity or other factors, *e.g.*, aging, rather than from exposure to genotoxic chemicals. Genotoxicity may contribute to many other toxicity because it can affect at early stage of the adverse outcome pathway of various endpoints. Generally other toxicity is visible, we can see the outcomes of toxicity and also suffer from such diseases. While outcomes of genotoxic effects are generally silent, such genotoxic damage can affect people indirectly, for example they might cause cancer, which affects patients much later. Therefore, genotoxicity testing, which can be engaged short-term and cheaply, was designed to identify potential carcinogenic chemicals, which is very time consuming and costly determine experimentally using lifetime cancer bioassays or human epidemiology. Following data accumulation and a greater understanding the mechanisms of carcinogenicity, it became less simple to make a direct connection between genotoxicity and carcinogenicity. The use of genotoxicity data for predicting carcinogens was diminished but re-directed to understanding mechanisms of carcinogenicity and also for evaluating heritable adverse effects to the next generation. The term “MCR: Mutagenicity, Carcinogenicity, Reproductive toxicity” has been introduced and become well known; it means these three endpoints are related but are independent events. Genotoxicity is important in regulatory science because it is often the key contributor to the understanding of carcinogenicity and other genetic diseases. It is also a key determinant of whether the subsequent disease outcome behaves as a threshold event, with levels of exposure that do not lead to the disease.

### Statistical evaluation of test data

Many researchers as well as regulatory people have used statistical comparison tests for evaluating genotoxicity test results. Statistical tests give the probability of the expected events occurring. Nevertheless, it is important to emphasize that statistical tests are based on many assumptions, for example, population distribution of the event; null and alternative hypothesis; nominal probability of errors (type I and type II); etc. Often, toxicologists do not pay sufficient attention to such assumptions. In the statistical comparison tests, the null hypothesis is that the data of the treatment group(s) are located inside the data distribution of the negative control group within a pre-determined *p*-value (*e.g.*, 0.05 or 0.01). In toxicological studies, if the data is not met the null hypothesis then we call the result is positive, although it means only that the data have not met the negative control distribution under the pre-set condition (not negative). Nominal type I and type II error rates should be fixed by experimenters or sometimes by regulatory persons according to the purpose of the test. Generally, people use 0.05 (one event occurs among 20 trials) or 0.01 (one event occurs among 100 trials) as the nominal type I error rate, however, such numbers have been based on the specialists’ intuition but not been established on a scientific basis. We just think one over 20 looks rare and one over 100 is a very rare case. Moreover, the type II error rate, which is detection power of the test, is often not taken into account. These *p*-values should be determined in advance of designing the particular assay. For example, in the in vivo micronucleus assay it should be determined in advance how many cells should be analyzed and how many animals should be assigned to each control and treatment group in order to achieve an optimal experimental condition [11]. It is most important that the “positive” outcome is defined in advance. The Ames assay used doubling as the positive definition without any detailed discussion on this point, and regulation used the judgement on this criterion for a long time. If we assume that a 10% increase from the negative control is positive, we must design the assay protocol using more animals or more cells should be analyzed.

### Biological relevance *vs* statistical significance

Another important factor for evaluating data is biological or toxicological relevance. This factor includes the dose–response relationship, the level of increase from the control (concurrent/historical), and reproducibility. These factors are often set based on the knowledge and experience of experts and originally such intuitional factors should be supported by statistics. We can understand and accept these approaches as a general idea, and therefore these intuitive guides appear less transparent and more subjective than the outcomes of statistical tests. Here, I compare the descriptions of OECD test guidelines (as an example referring TG-474, the in vivo micronucleus test) chronologically.

TG-474 adopted on 26 May 1983 said “*Data should be evaluated by using appropriate statistical methods…There are several criteria for determining a positive result, one of which is a statistically significant dose-related increase in the number of micronucleated polychromatic erythrocytes. Another criterion may be based upon detection of a reproducible and statistically significant positive response for at least one of the test points*” [12].

TG-474 adopted on 21st July 1997 said “*There are several criteria for determining a positive result, such as a dose-related increase in the number of micronucleated cells or a clear increase in the number of micronucleated cells in a single dose group at a single sampling time. Biological relevance of the results should be considered first. Statistical methods may be used as an aid in evaluating the test results* [9, 10]*. Statistical significance should not be the only determining factor for a positive response. Equivocal results should be clarified by further testing preferably using a modification of experimental conditions… A test substance for which the results do not meet the above criteria is considered non-mutagenic in this test*” [13].

TG-474 adopted on 29 July 2016 said “*Providing that all acceptability criteria are fulfilled, a test chemical is considered clearly positive if: a) At least one of the treatment groups exhibits a statistically significant increase in the frequency of micronucleated immature erythrocytes compared with the concurrent negative control, b) This increase is dose-related at least at one sampling time when evaluated with an appropriate trend test, and c) Any of these results are outside the distribution of the historical negative control data (e.g. Poisson-based 95% control limits). If only the highest dose is examined at a particular sampling time, a test chemical is considered clearly positive if there is a statistically significant increase compared with the concurrent negative control and the results are outside the distribution of the historical negative control data (e.g. Poisson-based 95% control limits)* [14]. *Recommendations for the most appropriate statistical methods can be found in the literature* [15–18]*.*

There are differences among these versions on description of data evaluation. The 1983 and the 2016 versions look rather similar and both suggested primarily statistical analyses for the data evaluation. The latest 2016 version provides a more precise description and recommends the appropriate statistical methods as references. In contrast, the 1997 version was different from the others. It recommended that biological relevance of the results should be considered first. It noted that statistical methods may be used for helping or supporting of evaluating the test results. Moreover, it concluded that statistical significance should not be the only determining factor for a positive response. I, personally, prefer the 1997 version to others because I believe biological relevance should be considered first using, if possible, a graphic image on which all data are plotted and understood at a glance. If we understand statistics well and design the experiment properly, and also apply the proper statistical test method, then, of course, we can appreciate the outcomes of statistical test to endorse biological relevancy.

### Qualitative evaluation and quantitative evaluation

At the early stage, even now, for establishing mutagenicity tests, for example, the Ames test and in vitro chromosomal aberration test using Chinese hamster lung cells, especially in Japan, we used simple criteria for calling the tests. We use a doubling of number of revertant colonies for the Ames test and equal to or more than 10% of cells analyzed having chromosomal aberrations. These criteria have been accepted by regulatory bodies as the proper judgement without any trouble for a long time.

Genotoxicity has been evaluated qualitatively but not quantitatively in many countries. In Japan, a little differently from other countries, we introduced semi-quantitative evaluation for the genotoxicity judgement for regulatory purposes. For example, we have used a doubling as a criterion for the Ames test and in addition we have used the mutation rate defined as number of revertant (rev) colonies per 1 mg (rev/mg) of a test material. We regard an agent as a strong mutagen if the rate is more than 1000 rev/mg; the Industrial Safety and Health Act requires an additional assay, for example, an in vitro chromosomal aberration test. In the Chemical Substance Control law, a chemical is classified as a designated chemical when showing more than 1000 rev/mg in the Ames test (nowadays, the law has been revised and the criterion is not used). In the in vitro chromosomal aberration assay, we have introduced the D_20_ value that was defined as the predicted concentration (µg/mL) at which 20% of analyzed metaphases cells had one or more chromosomal aberration. In the Chemical Substance Control law mentioned above, the chemical was classified as a designated chemical when the D_20_ value was 0.01 mg/mL or less (as same as the Ames test). Moreover, we frequently discussed using the D_20_ as an indicator of potential clastogenicity.

## Interpretation of chemical genotoxicity

As mentioned before, we regarded genotoxic chemicals as potential carcinogens based on the good correlation between genotoxic and carcinogenic outcomes at the first era. Pharmaceutical, agricultural, and other chemical developers stopped developing their chemicals when the Ames test was positive, due to concern about potential carcinogenic activity. The regulatory bodies also tended not to accept the release of such Ames positive chemicals into the market, especially chemical substances for direct human use. Then, after a lot of data were accumulated and showed that genotoxicity was not a prerequisite for all carcinogenicity, some chemicals without mutagenic potential were recognized to be carcinogenic and, vice versa, some Ames test positive chemicals did not show carcinogenic potential.

The Ames test has been most popular and is in widespread routine use. The in vitro chromosomal aberration test is also frequently performed, especially in Japan using Chinese Hamster Lung cells. The in vitro chromosomal aberration test has been shown to give positive results by some non-specific factors, such as osmolality and pH, that is by un-physiological culture conditions. Such positive results are not considered to be a genotoxic event because such extreme experimental conditions are unrealistic and not expected in humans in vivo. Some in vitro chromosomal aberration positive chemicals did not induce micronuclei as an indicator of chromosomal aberrations in erythropoietic cells in vivo. Hence the positive outcomes of the in vitro chromosomal aberration test are considered sometimes ambiguous for genotoxicity evaluation. As an example, the genotoxicity and carcinogenicity of pesticides evaluated from 2012 to 2016 by the Agricultural Chemicals Expert Committee of the Cabinet Office Food Safety Commission in Japan was analyzed [19]. The study aimed to see how much the in vitro chromosomal aberration test contributed to the final safety judgment of pesticide. A total of 183 chemicals were evaluated during this 5-year period and all have been approved as pesticides. More than 1/4 chemicals were positive in the in vitro chromosomal aberration test. And, as expected, there were few chemicals that showed a clear Ames test positive result because developers might not put in applications for Ames test positive chemicals. Moreover, surprisingly, although it was not the main theme of this manuscript, nearly half were positive in the long term carcinogenesis assay, in at least one species and one target organ.

The most important role for genotoxicity information in chemical safety assessment has been the determination of whether or not genotoxicity is a key initiating event in carcinogenesis. A carcinogen that is genotoxic, especially at the carcinogenesis target site, is regarded as a “genotoxic carcinogen” that has no threshold [20, 21]. This means under the current assessment strategy, genotoxic carcinogenicity is a key factor for chemical safety to human beings. It indicates, in this sense, the genotoxicity information plays an important role for the final regulatory judgement.

## Future—Conclusion and proposals

We have to think more deeply about the final aim of chemical genotoxicity information in human health risk assessment. If genotoxicity is exclusively important for identifying genotoxic carcinogens that have no threshold and not acceptable putting into market, then genotoxic assays are not needed for every chemicals, but only for carcinogenic chemicals. It is, however, true that the current long-term bioassay for carcinogenicity is time consuming and costly and not practical to perform on all chemicals being developed. Nevertheless, genotoxicity information alone does not determine human risk. For example, if a chemical was negative in the long-term carcinogenicity bioassay, showed a no observed-adverse-effect level greater than 1000 mg/kg/day, no reproductive adverse effect but showed positive result in the Ames test and the in vitro chromosomal aberration test, then how shall we treat it for human safety risk assessment? For the final decision, we have to take the expected benefit of the chemical for our lives into account to balance risk and benefit.

The most important feature of genotoxicity is the absence of a threshold. This is the reason why genotoxicity information is considered important because it is necessary to identify genotoxic carcinogens. Regulatory people tend to believe that genotoxicity does not have threshold. Nevertheless, data have been accumulated that show thresholds, at least apparent or biological thresholds even for direct DNA acting genotoxic chemicals. If we accept the existence of thresholds, then the level of exposure to humans becomes critical for assessment of safety risk. I believe it is time to stop discussion about existence of thresholds and to accept practical thresholds and to start considering levels of exposure for genotoxicity.

It is difficult to estimate chemical exposure to humans except for pharmaceutical drugs for human use. In human use pharmaceuticals drugs, we know the exposure level and benefit of the treatment, thus we can control the risk–benefit balance. Nevertheless, for example, in the case of industrial chemicals it is difficult to make human exposure assessments, because we generally do not know the volume of chemical that was released into our environment and how much reaches us through water, air and through other routes. And it is more difficult to quantify the benefit of the chemical at a given level of exposure. Because the actual quantity of the chemical we intake into our body is very small, I am curious whether we can assess safety of the chemical using existing test systems which evaluate their toxic effects using up to very high exposure levels and extrapolation to the actual human exposure levels. Outcomes of tests evaluated up to extremely high concentrations in in vitro and unrealistic high dose-levels in in vivo may be meaningless for extrapolation to the actual safety risk. I have to note this situation is not only for genotoxicity but also for other toxicology endpoints including carcinogenicity. Sometimes we see TV news on the serious adverse effects of carcinogens to humans. However, almost all these were the result of accidents, improper uses/handling, or exposures to naturally existing local contaminants like some heavy metals. Most of these occasions can be explained by the extremely high exposure levels in comparison to the actual situation.

Considering all, I would like to propose to change the strategy for chemical safety assessment from the current system that requires all information should be gathered according to the guideline before application to the regulatory body and then the safety assessment start using the full data set. It may be reasonable if we have enough resource, time and budget, but it is inefficient. ILSI/HESI proposed “Risk 21” [22–24], and several years later another report has been published [25]. The Risk 21 is based on a step by step approach for chemical safety evaluation. Firstly, “Problem formulation” is followed by “Estimation of human exposure”, “Estimate of human toxicity” and then evaluating the “Intersection of exposure and toxicity on a RISK21 matrix”. Then a judgement is made whether there is sufficient information to make a decision. If “YES”, then we make a conclusion but if “NO” we start again from the “Problem formulation” for a second cycle based on the results of the first one. We repeat the cycle until we have enough confidence to make a decision. This is a good idea for the evolution of the whole strategy. We should make decisions based on the minimum essential information/data we need, leading to the most efficient and accurate assessment. Nevertheless, we must consider that the assessment should be acceptable for regulatory purposes. Who, when, and how we engage each step is a key issue, so that sound conclusions are based on the essential facts that determine safety and risk. Such a new approach should be used not only for genotoxicity, but also for all kinds of expected toxicities. In other words, we have to make a rational assessment of risk based on relevant and sound information to avoid over- or under-estimation of risk.

## Data Availability

Not applicable.
